# Workplace Screening Identifies Clinically Significant and Potentially Reversible Kidney Injury in Heat-Exposed Sugarcane Workers

**DOI:** 10.3390/ijerph17228552

**Published:** 2020-11-18

**Authors:** Cecilia J. Sorensen, Lyndsay Krisher, Jaime Butler-Dawson, Miranda Dally, Lynn Dexter, Claudia Asensio, Alex Cruz, Lee S. Newman

**Affiliations:** 1 Center for Health, Work & Environment, Colorado School of Public Health, University of Colorado, Aurora, CO 80045, USA; lyndsay.krisher@cuanschutz.edu (L.K.); JAIME.BUTLER-DAWSON@cuanschutz.edu (J.B.-D.); MIRANDA.DALLY@cuanschutz.edu (M.D.); LYNN.DEXTER@cuanschutz.edu (L.D.); LEE.NEWMAN@cuanschutz.edu (L.S.N.); 2 Department of Emergency Medicine, University of Colorado School of Medicine, Aurora, CO 80045, USA; 3 Colorado Consortium on Climate Change and Human Health, University of Colorado, Aurora, CO 80045, USA; 4 Department of Environmental and Occupational Health, Colorado School of Public Health, University of Colorado, Aurora, CO 80045, USA; 5 Pantaleon, Guatemala City, Guatemala; cdeberger@pantaleon.com (C.A.); acruz@pantaleon.com (A.C.); 6 Department of Medicine, Division of Pulmonary Sciences and Critical Care Medicine, University of Colorado School of Medicine, Aurora, CO 80045, USA

**Keywords:** heat stress, kidney disease, agricultural workers, occupational interventions, Central America

## Abstract

An epidemic of chronic kidney disease of unknown origin (CKDu) has emerged in the past two decades in agricultural communities, characterized by progressive renal failure with a dearth of early clinical symptoms. The aim of this study is to improve understanding of the natural history of this disease and to evaluate the impact of an educational and behavioral intervention on the trajectories of renal decline among a cohort of Guatemalan sugarcane workers. We identified groups of workers based on their kidney function during a longitudinal parent study conducted among sugarcane workers during the 2016–2017 harvest season. At the study’s first time point in February 2017, workers who developed abnormal kidney function (AKF) (estimated glomerular filtration rate, eGFR, <60 mL/min per 1.73 m^2^) were placed in the AKF group, workers with reduced kidney function (RKF) (eGFR 60–89) were placed in the RKF group, and workers who maintained normal kidney function (NKF) (eGFR ≥ 90) were placed in the NKF group. As part of the study, a health promotion, behavioral and educational intervention centered on water, electrolytes, rest, and shade (WERS) was provided to all study participants. We then prospectively analyzed renal function at the three study time points in February, March, and April. Additional data collected from previous harvests allowed for retrospective analysis and we compared the rate of change in eGFR over the previous five years (2012 to 2016) for each identified group. Mixed effects linear regression with random intercepts for the workers was used to investigate the difference in rates of change for the three groups and to assess the impact of the intervention study on rate of change of kidney function during the study compared to each group’s prior trajectory, utilizing the retrospective data collected during the five years prior to the study intervention. Between 2012 and 2016, eGFR declined at a rate of 0.18 mL/min per 1.73 m^2^ per year for the NKF group (95% CI: −0.66, 0.29, *p* = 0.45), 2.02 per year for the RKF group (95% CI: 1.00, 3.03, *p* = 0.0001) and 7.52 per year for the AKF group (95% CI: 6.01, 9.04, *p* < 0.0001). All study groups stabilized or improved their trajectory of decline during the intervention. This study supports the need to institute WERS interventions and to include mid-harvest screening protocols and longitudinal tracking of kidney function among sugarcane workers at high risk of CKDu. Early detection of rapid kidney function decline combined with appropriate interventions hold promise for stopping or slowing progression of renal insufficiency among these workers.

## 1. Introduction

Over the past three decades, chronic kidney disease of unknown origin (CKDu) has emerged as a global epidemic with hot-spots identified among agricultural communities in Latin America [1,2,3,4], India [5,6], Sri Lanka [7], Egypt [8], and the United States [9,10]. In Central America, it has become a leading cause of death and hospitalization, particularly among young, male agricultural workers, and has also been shown to affect women and children residing in the same communities [11,12,13,14,15]. The cause of CKDu is likely multifactorial and a variety of possible causes are under investigation including exposure to agrochemicals, heavy metals and silicates, heat stress, dehydration, intake of fructose-rich soft drinks, use of non-steroidal anti-inflammatory drugs (NSAIDs), tobacco use, and nutritional and genetic factors [16,17,18,19]. In the face of heat stress and/or dehydration, kidneys may be more vulnerable to nephrotoxic agents [12,20,21]. Despite its emergence over 30 years ago, little is known about the natural history of disease, including the rate of onset, speed of progression, and whether or not recovery is possible.

CKDu is often diagnosed at a late stage due to a lack of overt early clinical signs and symptoms [22]. Simultaneously, the agricultural sector in affected regions is characterized by high rates of worker migration as well as job attrition, making at-risk workers and disease cases difficult to follow over sustained periods of time [12,23]. In the case of the sugarcane industry of Guatemala, the setting for this study, field workers are hired on a seasonal basis and may live in local coastal communities or be migrant harvest workers hired from highland communities, adding further complexity to understanding the earliest manifestations of this disease as well as its natural history.

Experts and governmental bodies alike have recommended strengthening surveillance systems capable of monitoring CKDu incidence and prevalence [24,25,26,27]. Such systems, particularly in Central America, are either under development or are nonexistent, making it difficult to determine both the true burden of disease or the utility of screening [28]. Strengthening surveillance systems will allow for the early identification of high-risk individuals. Benefits of such systems will depend, in part, on whether they can identify opportunities for preventing progression or reversing kidney injury.

Additionally, practical and evidence-based guidance to inform occupational health interventions once screening is complete are lacking [28]. Early intervention and modification of known CKDu risk factors may, theoretically, help to prevent and possibly reverse progression of CKDu. There have been very few intervention-based studies that focus on prevention of kidney injury [29]. To date, most interventions in Latin America have focused on reducing heat exposure, increasing the number of rest breaks, and improving hydration and electrolyte administration which have shown to improve markers of kidney function [12,22,30,31,32]. For example, Wegman et al. reported an attenuated decrease in cross-shift estimated glomerular filtration rate (eGFR) with an intervention that utilized an adapted water, rest, and shade (WRS) protocol [12] and Glaser et al. reported decreased incidence of incident acute kidney injury as well as a lower decline in cross-harvest eGFR with an intervention that altered rest breaks and improved access to shade and appropriate hydration [32]. Butler-Dawson et al. demonstrated that cross-shift incidence of acute kidney injury was associated with both dehydration and lower electrolyte supplementation [33]. In a related study, Sorensen et al. observed serum hyperosmolarity was associated with these cross-shift declines in kidney function, Sorensen et al. [34] an effect that may be regulated via a vasopressin-mediated pathway of renal injury [35]. Ideally, to show the long-term success of such interventions in preventing kidney disease, longitudinal screening data should be combined with well-implemented interventions and longitudinal follow up.

In order to improve the understanding of the natural history of renal disease in this occupational setting, we examined six years of data including health histories and renal function of a cohort of Guatemalan sugarcane workers. This included prospective data collected during the harvest for those employed during the 2016–2017 harvest, in combination with the prior five years of pre-employment medical data. Importantly, in the 2016–2017 harvest season, we prospectively evaluated the impact of a health protection, behavioral, and educational interventions on the trajectories of renal decline among this cohort of workers by comparing rates of pre-intervention kidney function decline to post-intervention decline. A better understanding of the natural history of this devastating disease serves to inform surveillance programs and interventions aimed at prevention, detection, and diagnosis of illness in the subclinical phase and to examine if early workplace interventions can halt or reverse renal injury.

## 2. Materials and Methods

### 2.1. Parent Study Design

The parent study has been described previously by Butler-Dawson et al. [33]. Briefly, the study was conducted across the 2016–2017 harvest season in a population of 517 field workers employed by Pantaleon, a sugarcane agribusiness in Guatemala. Workers were recruited into the study in January 2017. During the study period, laboratory data, including point-of-care (POC) creatinine, were collected before and after three work shifts for each study participant across three months: February, March, and April 2017 (one work shift per participant per month). One goal of the study was to assess the impact and feasibility of workplace practices aimed at preventing acute kidney injury by increasing hydration (electrolytes and water) in addition to structured rest and shade.

The sugarcane harvest season runs from November until May each year. All participants were over the age of 18 and had passed the pre-employment medical screening which required workers to have a pre-season eGFR of at least 60 mL/min per 1.73 m^2^. Workers consisted of sugarcane cutters, who cut and pile sugarcane, and production workers, who perform tasks such as cutting and planting cane seed. Production workers are from the local communities or “zona” and live in their own homes during the harvest. Cane cutters originate from both the zona and the highland communities or “altiplano”; altiplano workers live in dormitories at the mill for the duration of the harvest. Work setting and work practice details have been previously described [30].

The agribusiness has been implementing a water, rest, and shade program since 2009. At the time of the study, through this program, cane cutters were encouraged to drink 16 L of water and 2.5 L of electrolyte solution per work shift and to take three 20-min breaks and one 60-min lunch break during the work shift. Cane cutters were also encouraged to rest in tarp shade that was provided. The field health aides educated the workers on health topics including hydration, rest, and safety.

As part of the parent study, we built upon the current water, rest, and shade program. During the course of the study, workers received an enhanced WERS (water, electrolytes, rest, and shade) program that was modified from the agribusiness’ program. The WERS intervention included two components: (1) amplifying the existing worker education program on the importance of WERS, and (2) providing a “wellness incentive” based on workers’ hydration status at the start and end of the work shift. Study personnel and trained Pantaleon nurse aides provided additional education through face-to-face communication, posters, and pocket urine color charts (Human Hydration, LLC, Hampton, VA, USA) for self-evaluation of hydration status, translated to Spanish and adapted for low literacy. For the wellness incentive, all study participants were offered incentives on their three study days. The worker received tokens if he started the study work shift hydrated (pre-shift urinary specific gravity was ≤ 1.020) [36] or if he maintained or improved his hydration status across the work shift (<1% body weight loss) [37] If a worker was not considered hydrated, he was encouraged to hydrate and take more rest breaks. The workers entered tokens into a raffle for chances to win small non-monetary prizes (i.e., soap, towels, socks, soccer balls, etc.) at the end of each study day. More details of this intervention have been previously described [33].

Ethics review and approval for this study was granted by the Colorado Multiple Institutional Review Board (COMIRB) and in Guatemala by the Comité de Ética, Facultad de Medicina, Universidad Francisco Marroquin-Hospital Universitario Esperanza (COMIRB Protocol #: 16-1824).

### 2.2. Current Study Design

For this current study, we retrospectively assigned participants from the parent study’s 517 participants into three categories based on their pre-shift eGFR at the first study time point in February 2017. Participants who were found to have a February pre-shift eGFR less than 60 mL/min per 1.73 m^2^ were categorized into the abnormal kidney function group (AKF), participants with a pre-shift eGFR at or above 60 and less than 90 mL/min per 1.73 m^2^ were placed into the reduced kidney function group (RKF), and those who maintained a pre-shift eGFR value at or above 90 mL/min per 1.73 m^2^ were identified as a comparison group and are hereafter referred to as workers with normal kidney function (NKF). Any workers with missing February eGFR values (*n* = 8) or workers missing both March and April pre-shift eGFR measurements (*n* = 26) were excluded from the dataset, resulting in a total of 483 study participants (93%) for the current study. We examined differences between study workers included in this current analysis (*n* = 483) and study workers not included (*n* = 34) (Appendix A, Table A1). There were no significant differences in age (*p* = 0.21), race/ethnicity (*p* = 0.65), smoking (*p* = 0.55), hypertension (*p* = 0.39), alcohol intake (*p* = 0.51), water source (*p* = 0.53), pre-employment creatinine (*p* = 0.96), pre-employment eGFR (*p* = 0.41), or pre-employment BMI (*p* = 0.17). We did observe differences in home residence between the included workers and excluded workers (66% vs. 32% local workers, respectively, *p* < 0.01) and for job type between included versus excluded workers (80% vs. 94% cane cutters, respectively, *p* = 0.04). We also observed that those excluded due to missing both March and April timepoints did not have a significantly different February eGFR (*p* = 0.56).

### 2.3. Data Collection

**Figure A1:**
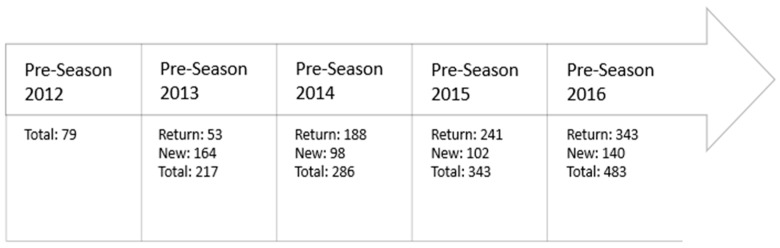
Work history of study participants.

Pre-employment medical screening (Retrospective data): Pantaleon annually collects and archives pre-employment survey, clinical, and laboratory data, including serum creatinine measures, as part of a pre-employment medical screening at the beginning of each harvest season. Data from the pre-harvest medical screenings were provided to the research team for the years 2012–2016 for the study participants. To construct the renal health histories of the workers in this study cohort, we utilized pre-employment data from 2012–2016. Serum creatinine was collected by venipuncture and sent to an independent, licensed clinical laboratory (Herrera Llerandi laboratory, Guatemala City, Guatemala). Additional clinical data included age, blood pressure (obtained after at least 3 min of seated rest before the measurement), weight, and height. Survey data included demographics, number of previous harvests worked, self-reported diabetes, home residence, smoking, alcohol use, drinking water source, and job type. Figure A1 shows the numbers of new and returning workers per year between 2012–2016.

Study pre-and post-shift sampling (Prospective data): POC creatinine was collected for each participant prior to the start of the work shift (5–9 a.m.) at three study time points (February, March, and April 2017) in addition to other laboratory parameters [33]. To measure creatinine pre- and post-shift, blood was collected by finger prick and read instantly in the field using the Nova^®^ Statscan (Stat Sensor Creatinine Meter, Nova Biomedical Corporation, Waltham, MA, USA). POC creatinine was repeated at the end of the work shift (1–4 pm for production workers and 4–7 pm for cane cutters). For this current analysis, we are only examining pre-shift POC creatinine. In previous studies conducted in similar worker populations in Guatemala, we have examined Nova^®^ Statscan meter reliably [38,39]. We have observed that there is good agreement between venous and capillary samples of pre-shift creatinine measurements, thus requiring no adjustment to relate capillary creatinine values to venous values taken prior to the start of work [38,39]. Pre-shift POC creatinine was used to calculate eGFR using the Chronic Kidney Disease Epidemiology Collaboration (CKD-EPI) equation for all participants setting race to “non-Black” [40]. Study sampling details are previously described [33].

Assessment of heat exposure: On each study day, we collected wet bulb globe temperature (WBGT) in the sugarcane field where the study group was working during work shift hours (3M QUESTemp 34, Thermal Environmental Monitor, St. Paul, MN, USA). WBGT is a measure of heat exposure and is used by the National Institute for Occupational Safety and Health (NIOSH) and the International Organization for Standardization (ISO) to establish guidelines for work/rest cycles in different environments [41]. The WBGT meter provided output on the average and maximum WBGT for each study day.

### 2.4. Additional Interventions and Education for Participants with Abnormal Kidney Function

**Figure 1:**
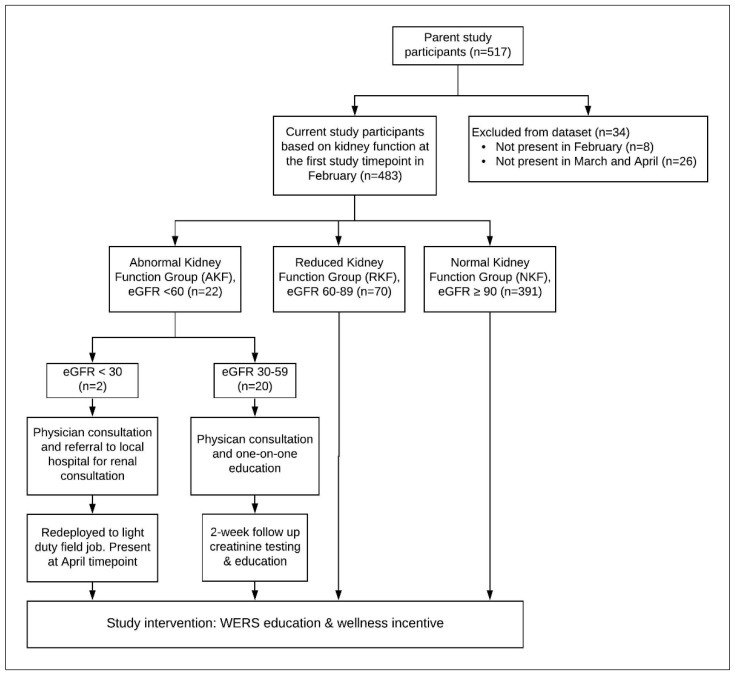
Study Design and Management of Study Screening Creatinine Outcomes. WERS: water, electrolytes, rest, and shade.

At the time of the study, the standard practice of the agribusiness was to screen sugarcane field workers pre-season and only allow those with an eGFR ≥ 60 mL/min per 1.73 m^2^ to be hired for field work. (Of note, the cutoff has since been increased to 90 mL/min per 1.73 m^2^.) By testing creatinine mid-season through the study time points, we identified workers who had dropped below their pre-harvest renal function. In addition to the study intervention, individual health interventions and education were provided to study workers in the AKF group by the Pantaleon medical staff (see Figure 1).

Among our current study cohort of 483 workers, those found to have a pre-shift eGFR of less than 30 mL/min per 1.73 m^2^ (AKF group, *n* = 2) at the February timepoint were immediately referred to the on-site medical clinic for consultation with a general medical doctor to take a medical history, perform a physical exam, recheck serum creatinine, and facilitate consultation with a renal specialist in Guatemala City for further work up and testing. These workers were redeployed to a light duty job and were retained in our study cohort. Workers found to have an eGFR at or above 30 and less than 60 mL/min per 1.73 m^2^ (AKF group, *n* = 20) in February were also immediately referred to the Pantaleon clinic for consultation with a doctor for clinical assessment. Other causes of renal failure were ruled out and the patient was given individual education on WERS, nutrition, and how to reduce exposure to other nephrotoxic agents, such as NSAIDS, tobacco, and alcohol. Workers’ serum creatinine levels were rechecked two weeks after the initial visit and further counseling was conducted. The cost of transportation to the Pantaleon clinic, the renal specialist in Guatemala City if applicable, as well as wages for lost work were compensated by Pantaleon.

Workers found to have an eGFR at or above 60 and less than 90 mL/min per 1.73 m^2^ (RKF group, *n* = 70) continued receiving the study protocol WERS education and wellness incentive that all workers participating in the parent study received.

### 2.5. Statistical Analysis

Retrospective analysis: Baseline characteristics of the three identified study groups (AKF, RKF, NKF) were based on the first harvest for which data were available (2012–2016) and were compared using chi-square or Fisher Exact tests for categorical characteristics; t tests with Satterthwaite corrections for unequal variances were used for continuous variables. The primary outcome variable was the rate of change in eGFR from 2012 to 2016. To investigate the difference in rates of change for AKF, RKF, and NKF groups, mixed effects linear regression with random intercepts for subjects were used. All models controlled for age at baseline, eGFR at baseline, and home residence (altiplano v. zona). We assessed the correlation of age at baseline and number of previous harvests worked (*r* = 0.50) and used age at baseline as a control variable in lieu of number of harvests worked.

Prospective analysis: To determine how the study and additional interventions (i.e., WERS, health, behavioral and educational) may have impacted rate of change of kidney function during the 2016–2017 harvest, mixed effects linear regression with random intercepts for subjects were also used. Models were controlled for 2016 pre-employment medical screening data including age, body mass index (BMI), mild hypertension (defined as systolic blood pressure = 130–139 mmHg and/or diastolic blood pressure = 80–89 mmHg), and home residence. Monthly rate of change in pre-shift eGFR from pre-employment to the start of the study (Aug-Feb) was compared to monthly rate of change in pre-shift eGFR during the study intervention (Feb-Apr) for each of the three groups using a three-way interaction term of group by month by pre- to post-intervention. Data were analyzed with SAS version 9.4 (SAS Institute Inc., Cary, NC, USA).

## 3. Results

### 3.1. Current Study Participants

Among the cohort of 483 workers, we identified 22 workers (5%) with a pre-shift eGFR below 60 mL/min per 1.73 m^2^ (AKF) and 70 workers (14%) with a pre-shift eGFR at or above 60 and less than 90 mL/min per 1.73 m^2^ (RKF) at the first study time point in February. There were 391 workers (81%) who maintained a pre-shift eGFR value at or above 90 mL/min per 1.73 m^2^ in February (NKF).

### 3.2. Heat Exposure

During the February study days, the average WBGT ranged from 29.5 to 32.9 °C and the maximum WBGT ranged from 31.7 to 36.4 °C. During March days, the average WBGT ranged from 28.2 to 32.2 °C and the maximum WBGT ranged from 31.2 to 35.5 °C. During the April days, the average WBGT ranged from 30.9 to 32.0 °C and maximum WBGT ranged from 33.5 to 35.5 °C. To put into context, the Occupational Safety and Health Administration (OSHA) heat exposure threshold for work/rest regimens for “very heavy” work at 30.0 °C is for workers to work 25% and rest 75% per hour [21,41]. Only 4 out of the 23 study days had an average WBGT below 30.0 °C.

### 3.3. Retrospective Assessment: Baseline Harvest Characteristics

Baseline demographic and clinical characteristics of the 483 workers are shown in Table 1. Workers identified in the NKF group had the highest mean pre-harvest baseline eGFR at 114.8 mL/min per 1.73 m^2^ (standard deviation [SD]: 13.3) compared to 102.2 mL/min per 1.73 m^2^ (SD: 17.4) for those in the RKF group and 102.6 mL/min per 1.73 m^2^ (SD: 18.4) in the AKF group (*p* < 0.01). Workers in the RKF group were on average older (33.5 years, SD: 8.7) compared to NKF and AKF groups (27.7 years [SD: 8.2] and 29.2 years [SD: 6.7], respectively, *p* < 0.01). A higher number of workers in the RKF and AKF groups were local workers (81.4% and 86.4%, respectively) compared to 62.2% local workers in the NKF group, *p* < 0.01. Cane cutters made up 79.9% of the study population, with similar representation in each of the three work groups. No differences were seen between groups for BMI, with a mean value of 23.1 kg/m^2^ (SD: 2.6). A higher proportion of workers in the RKF and AKF groups reported smoking (18%) and alcohol use (12%) compared to those in the NKF group (6.8% and 5.6%, respectively). Due to the small number of workers reporting these behaviors, smoking and alcohol use were not evaluated further.

### 3.4. Retrospective Assessment: Change in eGFR from 2012 to 2016

After controlling for baseline age, baseline eGFR, and home residence, the average yearly decline in eGFR from 2012–2016 for the NKF group was 0.18 mL/min per 1.73 m^2^ (95% CI: −0.66, 0.29, *p* = 0.45). Significant greater yearly declines were seen in eGFR for the two other groups at 2.02 mL/min per 1.73 m^2^ per year for the RKF group (95% CI: 1.00, 3.03, *p* = 0.0001) and 7.52 mL/min per 1.73 m^2^ per year for the AKF group (95% CI: 6.01, 9.04, *p* < 0.0001) (Table 2).

**Figure 2:**
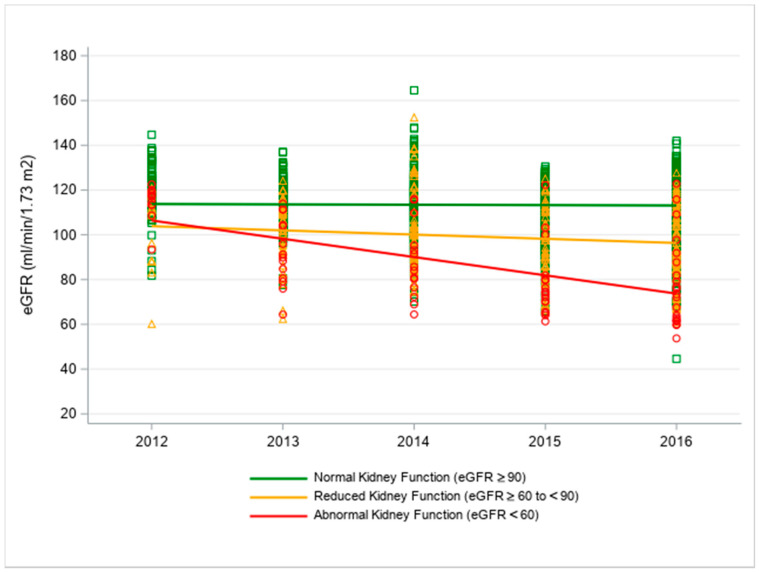
Fitted regression line showing annual change in eGFR values between kidney function groups, 2012–2016 harvest seasons.

Significant differences were seen between groups for the average yearly rate of decline in eGFR in the retrospective analyses. The interactive effect of group by year for change in eGFR is shown in Table 3. On average, the AKF group’s eGFR declined 7.34 mL/min per 1.73 m^2^ more per year compared to the NKF group (95% CI: 5.76, 8.93, *p* < 0.0001) and 5.51 mL/min per 1.73 m^2^ more per year compared to the RKF group (95% CI: 3.69, 7.33, *p* < 0.0001) (Table 3, Figure 2). A smaller, yet significant, yearly rate of decline was also observed for RKF versus NKF workers at 1.83 mL/min per 1.73 m^2^ (95%CI: 0.71, 2.95, *p* = 0.0014).

### 3.5. Prospective Assessment: 2016 Pre-Harvest Characteristics

Table 4 presents 2016 pre-harvest demographic and clinical characteristics of the 483 workers. Mean age of workers in the 2016 harvest for RKF workers was (36.0 years, SD: 8.8) compared to those in the NKF group (29.7 years, SD: 8.4) and the AKF group (32.2 years, SD: 6.5) group (*p* < 0.0001). The NKF group had the highest 2016 mean pre-harvest eGFR at 115.4 mL/min per 1.73 m^2^ (SD: 12.8) versus 96.9 mL/min per 1.73 m^2^ (SD: 16.4) for those in the RKF and 77.3 mL/min per 1.73 m^2^ (SD: 19.5) in the AKF groups (*p* < 0.0001).

### 3.6. Prospective Assessment: Effect of WERS Intervention on Trajectory of Renal Function Decline during the 2016–2017 Harvest Season

**Figure 3:**
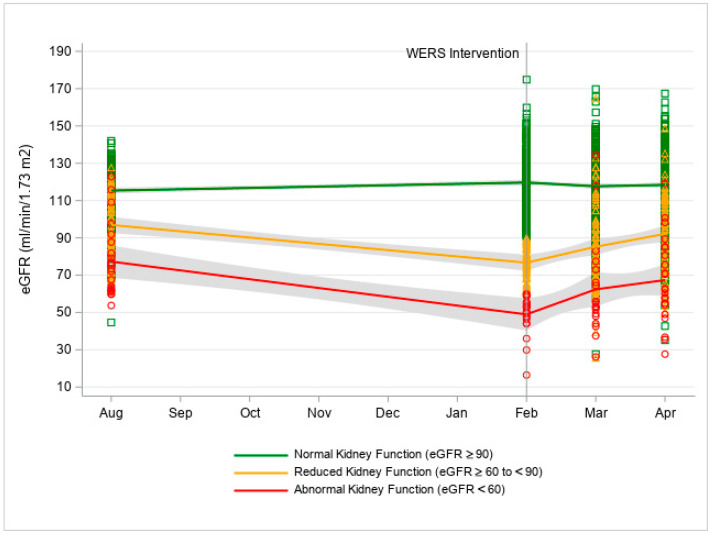
Loess line graph of change in pre-shift eGFR by kidney function group, pre- and post-WERS intervention during 2016–2017 harvest season.

Among the workers in the NKF group, average pre-shift eGFR was maintained during the pre-intervention period (Aug to Feb) and during the intervention period (February–April), with observed rates of change of 0.71 mL/min/1.73 m^2^ per month (95% CI: 0.41, 1.02, *p* < 0.0001) and 1.31 mL/min/1.73 m^2^ per month (95% CI: −0.61, 3.23, *p* = 0.181), respectively (Table 5 and Figure 3).

For RKF workers, declines were observed in the pre-intervention timeframe with improvements seen during the intervention. The average rate of decline in pre-shift eGFR for workers in the RKF group from pre-intervention to the start of the intervention was −3.36 mL/min/1.73m^2^ per month (95% CI: −4.08, −2.64, *p* < 0.0001). During the intervention months, pre-shift eGFR improved at an average rate of change of 6.05 mL/min/1.73 m^2^ per month (95% CI: 1.55, 10.54, *p* = 0.008). Overall, among the 70 workers in the RKF group, 52 (74%) experienced improvement in eGFR. Among the 52 workers, 37 had values above 90 mL/min/1.73 m^2^ and 15improved but stayed within 60–90 mL/min/1.73 m^2^ at their last study time point.

Similar results were observed for working in the AKF group. Workers in the AKF group were found to have an average rate of pre-shift eGFR decline of −4.37 mL/min per 1.73 m^2^ per month from pre-intervention to the start of the intervention (95% CI: −5.68, −3.06, *p* < 0.0001). During intervention months (February to April), the average pre-shift eGFR for workers in the AKF group changed at a rate of 5.62 mL/min per 1.73 m^2^ per month, although this was not statistically significant (95% CI: −2.65, 13.89, *p* = 0.183). Overall, among the 20 workers in the AKF group, 17 workers (85%) showed improvement in eGFR. Among the 17 workers, 10had values within 60–90 mL/min/1.73 m^2^ at and three of the 17 workers had eGFR values above 90 mL/min/1.73 m^2^ their last study timepoint. Four workers improved their eGFR but remained less than 60 their last study timepoint.

**Figure A2:**
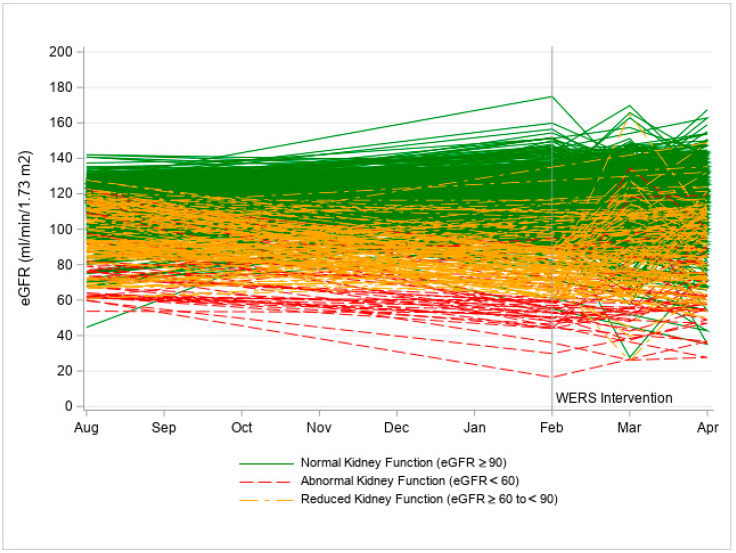
Change in eGFR, all subjects, by subject group, pre- and post-WERS intervention during 2016–2017 harvest season.

When comparing the rate of change in pre-shift eGFR pre-intervention to post-intervention, we observed that workers in the AKF and RKF groups, on average, showed significant improvement in rate of eGFR change after the intervention was implemented. That rate of change for the AKF improved by 9.99 mL/min per 1.73 m^2^ per month (95% CI: 1.62, 18.36, *p* = 0.02) while workers in the RKF had an improvement in rate of change of 9.41 mL/min per 1.73 m^2^ per month (95% CI: 4.85, 13.96, *p* < 0.0001). Workers in the NKF remained stable. Changes in eGFR values by individual participant by study group are illustrated in the Appendix A, Figure A2.

## 4. Discussion

This is the longest retrospective longitudinal study to date of renal function in workers at risk of CKDu and the first to examine the potential benefits of an intervention on long-standing trajectories of renal decline among workers. These findings provide a more complete picture of the natural history of the disease and support the value of enhanced public health interventions. In this study, a small but significant percent of workers who had an eGFR ≥ 90 mL/min per 1.73 m^2^ in August 2016 developed significant reductions in kidney function during the harvest season. Mid-season screening proved valuable in identifying the subgroups that had progressed to RKF and AKF. Retrospective analysis of five years of prior renal function data permitted reconstruction of the natural history of the decline in these groups. In hindsight, those identified as having RKF or AKF during the study period had been on a multi-year trajectory of decline, although still within the normal range of eGFR, preceding the incident discovery of renal function abnormalities during the study period. This finding suggests that the current screening guidelines which rely on a single pre-employment measure of kidney function are not sufficiently to identify and protect workers who have progressive underlying renal disease which appears to take several years to develop.

The value of occupational health screening depends, in part, on whether there are interventions that can halt or reverse clinical progression. The renal function of workers in the study appeared to benefit from the study WERS intervention and additional health, behavioral, and educational interventions. The workers in the RKF group who were found to have an eGFR between 60–89 mL/min per 1.73 m^2^ in February were shown to improve their renal function with WERS behavioral and educational interventions, when compared to their trajectories of decline before the intervention. Workers found to have an eGFR < 60 mL/min per 1.73 m^2^ in February also showed reversal of a trajectory of decline after the intervention was implemented. Workers who maintained normal kidney function before and during the season maintained kidney function pre- and post-intervention. However, since there was no group in our study that did not receive the intervention we cannot confirm whether the intervention contributed to the stable kidney function throughout the harvest. The success of this intervention among workers with reduced and abnormal kidney function suggests that efforts to increase hydration (as well as rest) may be important in slowing kidney decline among workers [26].

To date, there have been few other intervention-based studies in Latin America, [29] with most focused on similar efforts to reduce heat exposure, increase the number of rest breaks, and improve hydration [12,22,30,31,32]. Our results are consistent with prior studies that have shown an attenuated decline in average cross-shift and cross-season decline in eGFR with interventions aimed at reducing heat stress and dehydration [12,32]. This positive response suggests that with early identification and appropriate management, some workers with limited underlying kidney dysfunction may be able to return to work, with close clinical monitoring, if renal-protective WERS protocols, coupled in some cases with lighter work duties, are implemented in this working population.

Notably, our interventions included an increased focus on consumption of electrolyte solution as well as water, educational emphasis on reducing heat stress and on the recognition and avoidance of nephrotoxins, as well as enhanced clinical monitoring for workers identified with abnormal kidney function during the season. Contrary to the commonly held belief that CKDu progresses inexorably to end stage renal disease, this study suggests that early detection and intervention may either prevent or at least delay progression and warrants further study. Given the current scale of the epidemic, earlier identification of patients at risk of disease is critical. Some worker appeared to improve their kidney function throughout intervention period, however it is not clear if this actually represents a possible reversal of disease.

Our study identified two novel ways to identify workers at risk for CKDu earlier in the course of disease through strategic screening. There is mounting evidence that intermittent episodes of acute kidney injury preceded the onset of CKDu and contribute to its development [2,33,42,43]. Additionally, there is strong evidence that even asymptomatic workers suffer injury [2,26]. Indeed, multiple studies have documented significant cross-shift increases in serum creatinine and decrease in eGFR as well as urinary markers of injury in asymptomatic patients [2,26]. Our patients were identified during screenings conducted before the start of a work shift at random time points during the harvest season. Regardless of whether these episodes represent pre-renal dehydration, elevation of creatinine due to muscle breakdown, an acute (reversible) kidney injury or irreversible renal dysfunction, workers in this study who were found to have renal dysfunction at random timepoints were discovered to be on a long-standing trajectory of renal decline, and therefore likely had significant underlying sub-clinical renal disease. This suggests that in addition to acute kidney injury contributing to CKDu, it is also an important risk indicator which arises during challenge to the organ during demands imposed by high internal and external temperatures coupled with physical strain and dehydration, which are extremely common in this occupational setting [2,12,33,44,45]. Therefore, the finding of any degree of renal dysfunction may be the most reliable and early screening tool for identifying patients at risk of progressing to chronic kidney disease.

Knowing this, we can strategically implement screening protocols to identify these asymptomatic workers before their renal condition worsens. Currently, most large agrobusinesses in CKDu endemic areas conduct a health screening for workers before the harvest. Given that cyclic fluctuations in kidney function have been described in at-risk populations, [46] and that those with sub-clinical underlying renal function appear to become “clinical” when faced with multiple physiologic stressors during the work season, we strongly recommend mid-season screening for all workers employed in agricultural industries when working conditions involve exposure to high ambient temperatures coupled with high physical workloads. The ideal screening schedule cannot be deduced from the current data. However, to balance a pragmatic approach with the need to identify at-risk workers as soon as possible, we suggest that each worker receive a mid-harvest creatinine check at least twice during the harvest period. This screening should be coupled with ongoing WERS programs and education for all workers in addition to close medical supervision for workers identified as having possible underlying renal disease or other identified risk factors such as tobacco use, hypertension, and nephrotoxic medication use [23,33]. Longitudinal worker screenings can also be used to identify at-risk workers. As demonstrated in this study, workers at-risk for developing renal dysfunction display a multi-year decline prior to displaying gross laboratory abnormalities. Therefore, knowing the slope of decline, rather than an absolute laboratory renal function value, is most important in recognizing patients in the earliest stages of disease.

Similar to Gonzalez-Quiroz et al., we identified three cohorts of study participants who had discrete trajectories of decline in kidney function and in similar proportions [46]. However, in the Nicaraguan community-based cohort analyzed by Gonzalez-Quiroz et al., the rapid declining group dropped at a rate of 18.2 mL/min per 1.73 m^2^ per year, which is much faster compared to our rapidly declining group who dropped at a rate of 7.52 mL/min per 1.73 m^2^ per year. Our findings are more consistent with Wijkstrom et al. [47], who reported a rate of decline in eGFR of 7.0 mL/min per 1.73 m^2^ per year in Nicaraguan men, however, their cohort of study participants had pre-established CKDu, whereas our cohort had no clinical evidence of disease and had normal eGFR within 6 months of the onset of reduced kidney dysfunction. Based on these studies, it is clear that individuals with underlying renal dysfunction who are at-risk for CKDu decline at significant rates; meanwhile, roughly 80% of the at-risk population does not show decline. Based on available evidence, an annual rate of decline of greater than 4 mL/min per 1.73 m^2^ per year, should be set as the threshold to trigger medical or public health intervention, if we aim to be as sensitive as possible while still remaining pragmatic.

As in all longitudinal studies, loss to follow up can introduce “healthy-worker” selection bias. Likely, the most ill workers dropped out of the workforce and thus are not represented in this study. In the present study, we were only able to capture workers who had an eGFR high enough to be hired and who stayed healthy enough to continue the job until our study started in February 2017, three months after hire. We also used a linear assumption to calculate the rate of decline in eGFR when in fact there is evidence to support a non-linear or step-wise rate of decline among CKD patients [48]. With the current data available for this study, we were unable to visualize more discrete granularity in the retrospective analysis and thus favored use of a linear assumption. Additionally, limited number of workers within the AKF caused wide confidence intervals for some of our effect estimates and limited our statistical power. While we were able to uncover varying degrees of renal dysfunction, we were unable to obtain follow up on patients in the cohort to see which individuals may have later progressed to develop CKDu after the harvest season ended. We were also unable to obtain if the success of the intervention led to lasting changes in the overall rate of decline of workers with renal dysfunction. With the use of POC creatinine meters, we caution that the reliability may not be applicable in all settings. We only tested specimens under a narrow range of temperature and humidity. Further studies are needed to determine if this formula is generalizable to other field environments, including wider ranges of temperature and humidity. We elected to track pre-shift eGFR during the intervention period as post-shift eGFR may be more susceptible to be influenced by acute dehydration and muscle breakdown. However, we cannot exclude the possibility that even pre-shift eGFR could be influenced by chronic dehydration that could be made worse by sleeping in warm temperatures at night. Our data emphasize the need for such prospective studies to be performed.

## 5. Conclusions

This study supports the need to institute WERS interventions and to include mid-harvest screening protocols and longitudinal tracking of kidney function among workers employed in occupations at high risk of CKDu. Early detection of rapid kidney function decline combined with appropriate interventions hold promise for stopping or slowing progression.

## Figures and Tables

**Table 1:** Baseline * Pre-Harvest Demographic and Clinical Characteristics by Assigned Kidney Function Group.

Characteristics	All Workers (*n* = 483)	AKF Workers (*n* = 22)	RKF Workers (*n* = 70)	NKF Workers (*n* = 391)	
	Mean (SD)	Mean (SD)	Mean (SD)	Mean (SD)	*p* Value
Age, years	28.6 (8.5)	29.2 (6.7)	33.5 (8.7)	27.7 (8.2)	<0.0001
Number of harvests worked	2.9 (1.2)	3.5 (1.3)	3.1 (1.3)	2.8 (1.2)	0.01
Body mass index, kg/m^2^ (*n* = 320)	23.1 (2.6)	23.4 (2.6) (*n* = 16)	23.8 (2.7) (*n* = 49)	23.0 (2.5) (*n* = 255)	0.11
eGFR, ml/min per 1.73 m^2^	112.38 (15.02)	102.6 (18.4)	102.2 (17.4)	114.8 (13.3)	<0.0001
**Race/Ethnicity, *n* (%) (*n* = 448)**					
Latino	137 (30.6%)	3 (15.0%) (*n* = 20)	24 (36.4%) (*n* = 66)	110 (30.4%) (*n* = 362)	0.19
Indigenous	311 (69.4%)	17 (85.0%)	42 (63.6%)	252 (69.6%)	
**Home residence, *n* (%)**					
Local (Zona)	319 (66.1%)	19 (86.4%)	57 (81.4%)	243 (62.2%)	0.0009
Highland (Altiplano)	164 (34.0%)	3 (13.6%)	13 (18.6%)	148 (37.9%)	
**Job Type, *n* (%)**					
Cane Cutter	386 (79.9%)	19 (86.4%)	59 (84.3%)	308 (78.8%)	0.42
Production Worker	97 (20.1%)	3 (13.6%)	11 (15.7%)	83 (21.2%)	
Mild hypertension ** *n* (%) (*n* = 243)	120 (49.4%)	8 (57.1%) (*n* = 14)	14 (34.2%) (*n* = 41)	98 (52.1%) (*n* = 188)	0.09
Smoking, *n* (%) (*n* = 317)	29 (9.2%)	3 (18.8%) (*n* = 16)	9 (18.4%) (*n* = 49)	17 (6.8%) (*n* = 252)	0.01 ***
Alcohol intake, *n* (%) (*n* = 317)	22 (6.9%)	2 (12.5%) (*n* = 16)	6 (12.2%) (*n* = 49)	14 (5.6%) (*n* = 252)	0.11 ***

* Baseline is defined as the first-recorded harvest worked between the years 2012–2016. ** Mild hypertension: systolic blood pressure = 130–139 mmHg and/or diastolic blood pressure = 80–89 mmHg. *** Fisher’s Exact Test.

**Table 2:** Mixed effects linear regression analyses showing yearly rate of decline in eGFR by kidney function group, 2012–2016 harvest seasons, N = 479 *.

Effect	Estimate (95% CI)	*p* Value
NKF (*n* = 387)	−0.18 (−0.66, 0.29)	0.451
RKF (*n* = 70)	−2.02 (−3.03, −1.00)	0.0001
AKF (*n* = 22)	−7.52 (−9.04, −6.01)	<0.0001

* Models controlled for baseline age, baseline eGFR, and home residence.

**Table 3:** Mixed effects linear regression analyses showing annual change in eGFR values between kidney function groups, 2012–2016 harvest seasons, N = 479 *.

Group Comparisons	Rate of Yearly Change in eGFR, Between Group Comparisons	
NKF, *n* = 387	Effect (95% CI)	*p* value
RKF, *n* = 70		
AKF, *n* = 22		
RKF vs. NKF	−1.83 (−2.95, −0.71)	0.0014
AKF vs. NKF	−7.34 (−8.93, −5.76)	<0.0001
AKF vs. RKF	−5.51 (−7.33, −3.69)	<0.0001

* Models controlled for baseline age, baseline eGFR, and home residence.

**Table 4:** 2016 Pre-Harvest Demographic and Clinical Characteristics by Assigned Kidney Function Group.

Characteristics	All Workers (*n* = 483)	AKF Workers (*n* = 22)	RKF Workers (*n* = 70)	NKF Workers (*n* = 391)	
	Mean (SD)	Mean (SD)	Mean (SD)	Mean (SD)	*p* Value
Age, years	30.7 (8.7)	32.2 (6.5)	36.0 (8.8)	29.7 (8.4)	<0.0001
Body mass index, kg/m^2^ (*n* = 448)	23.2 (2.5)	22.8 (1.5) (*n* = 20)	23.5 (2.5) (*n* = 66)	23.1 (2.6) (*n* = 362)	0.46
Creatinine, mg/dL	0.90 (0.17)	1.27 (0.22)	1.00 (0.17)	0.86 (0.12)	<0.0001
eGFR, ml/min per 1.73 m^2^	110.9 (16.8)	77.3 (19.5)	96.9 (16.4)	115.4 (12.8)	<0.0001
Mild hypertension *, *n* (%) (*n* = 448)	154 (34.4%)	5 (25.0%) (*n* = 20)	29 (43.9%) (*n* = 66)	120 (33.2%) (*n* = 362)	0.16
Smoking, *n* (%) (*n* = 444)	48 (10.8%)	4 (20%) (*n* = 20)	6 (9.1%) (*n* = 66)	38 (10.6%) (*n* = 358)	0.37
Alcohol intake, *n* (%) (*n* = 444)	41 (9.2%)	3 (15.0%) (*n* = 20)	6 (9.1%) (*n* = 66)	32 (8.9%) (*n* = 358)	0.66
**Water Source at Home, *n* (%), (*n* = 447)**					
Municipal	345 (77.2%)	13 (65.0%)	55 (84.6%)	277 (76.5%)	0.19 **
Well	86 (19.2%)	6 (30.0%)	10 (15.4%)	70 (19.3%)	
Surface Water	16 (3.6%)	1 (5.0%)	0 (0.0%)	15 (4.1%)	

* Mild hypertension: systolic blood pressure =130–139 mmHg and/or diastolic blood pressure = 80–89 mmHg. ** Fisher’s Exact Test.

**Table 5:** Mixed effects linear regression analysis of cross-season changes in eGFR values by kidney function group in the 2016-2017 harvest season, N = 448 *.

Rate of Change in eGFR	NKF (*n* = 362)	*p* Value	RKF (*n* = 66)	*p* Value	AKF (*n* = 20)	*p* Value
Pre-intervention (August 2016–February 2017), ml/min/1.73m^2^ (95% CI)	0.71 (0.41, 1.02)	<0.0001	−3.36 (−4.08, −2.64)	<0.0001	−4.37 (−5.68, −3.06)	<0.0001
Post-intervention (February–April 2017), ml/min/1.73m^2^ (95% CI)	1.31 (−0.61, 3.23)	0.181	6.05 (1.55, 10.54)	0.008	5.62 (−2.65, 13.89)	0.183
Difference in monthly rate of eGFR change between pre-intervention and post-intervention (95% CI)	0.60 (−1.34, 2.54)	0.548	9.41 (4.85, 13.96)	<0.0001	9.99 (1.62, 18.36)	0.019

* Models controlled for 2016 pre-harvest age, BMI, mild hypertension, and home residence. Missing blood pressure data on 35 workers. Mild hypertension: systolic blood pressure = 130–139 mmHg and/or diastolic blood pressure = 80–89 mmHg.

## Appendix A

**Table A1:** Comparison of 2016 pre-employment data for workers excluded from current study due to missing eGFR values in Febuary 2017 or missing both March and April 2017 values.

**Characteristics**	**All Workers (*n* = 517)**	**Excluded Workers (*n* = 34)**	**Included Workers (*n* = 483)**	***p* Value**
	**Mean (SD)**	**Mean (SD)**	**Mean (SD)**	
Age, years	30.6 (8.6)	28.8 (7.9)	30.7 (8.7)	0.21
Number of harvests worked	2.9 (1.2)	2.8 (1.4)	2.9 (1.2)	0.68
Body mass index (*n* = 478)	23.2 (2.5)	23.8 (2.7) (*n* = 30)	23.2 (2.5) (*n* = 448)	0.17
Creatinine, mg/dL	0.90 (0.16)	0.90 (0.12) (*n* = 33)	0.90 (0.17) (*n* = 483)	0.96
eGFR, ml/min per 1.73 m^2^	111.1 (16.7)	113.4 (14.2) (*n* = 33)	110.9 (16.8) (*n* = 483)	0.41
**Race/Ethnicity, *n* (%), (*n* = 478)**				
Latino	145 (30.3%)	8 (26.7%)	137 (30.6%)	0.65
Indigenous	333 (69.7%)	22 (73.3%)	311 (69.4%)	
Home residence, *n* (%)				
Local (Zona)	330 (63.8%)	11 (32.4%)	319 (66.1%)	<0.0001
Highland (Altiplano)	187 (36.2%)	23 (67.7%)	164 (33.9%)	
**Job Type, *n* (%)**				
Cane Cutter	418 (80.9%)	32 (94.1%)	386 (79.9%)	0.04
Production Worker	99 (19.2%)	2 (5.9%)	97 (20.1%)	
Smoking, *n* (%) (*n* = 473)	52 (11.0%)	4 (13.8%) (*n* = 29)	48 (10.2%) (*n* = 444)	0.55 **
Mild hypertension *, *n* (%) (*n* = 478)	162 (33.9%)	8 (26.7%) (*n* = 30)	154 (34.4%) (*n* = 448)	0.39
Alcohol intake, *n* (%) (*n* = 474)	45 (9.5%)	4 (13.3%) (*n* = 30)	41 (9.2%) (*n* = 444)	0.51 **
**Water Source, *n* (%) (*n* = 477)**				
Municipal	369 (77.4%)	24 (80.0%)	345 (77.2%)	0.53
Well	90 (18.9%)	4 (13.3%)	86 (19.2%)	
Surface Water	18 (3.8%)	2 (6.7%)	16 (3.6%)	
**Comparison of 2017 February Pre-Shift eGFR for Excluded Workers Missing Both March and April eGFR Values and Included Workers with March and/or April eGFR Values. *****				
	**All Workers (*n* = 509)**	**Excluded workers (*n* = 26)**	**Included Workers (*n* = 483)**	***p* Value**
	**Mean (SD)**	**Mean (SD)**	**Mean (SD)**	
February eGFR, ml/min per 1.73 m^2^	110.3 (24.7)	113.1 (30.7)	110.2 (24.4)	0.56

* Mild hypertension: systolic blood pressure = 130–139 mmHg and/or diastolic blood pressure = 80–89 mmHg. ** Fisher’s Exact Text. *** Missing 8 values from cohort of 517 study workers with no 2017 February morning eGFR data.
